# Influence of body shape on health-related quality of life in Korean adults: The mediating effect of self-rated health

**DOI:** 10.1371/journal.pone.0293286

**Published:** 2023-10-30

**Authors:** Eun Sil Her, Jung Kyu Park, Yun Kyoung Oh

**Affiliations:** 1 Department of Food and Nutrition, Changshin University, Changwon-si, Korea; 2 Department of Computer Engineering, Changshin University, Changwon-si, Korea; 3 Department of Consmetology, Changshin University, Changwon-si, Korea; Taipei Medical University, TAIWAN

## Abstract

A Body Shape Index (ABSI) predicts mortality independent of body mass index and had a negative relationship with self-rated health (SRH), which had a positive effect on the EuroQol (EQ)-5D index. This study aimed to investigate the relationship between ABSI and the EQ-5D index and to verify the mediating effect of SRH in Korean adults. This study included 13,381 participants aged ≥20 years from the 7th (2016-2018) Korean National Health and Nutrition Examination Survey (KNHANES). The participants were classified into tertile groups based on the ABSI (T1, T2, and T3 groups). Demographic characteristics, health-related factors, ABSI, SRH, and EQ-5D scores were evaluated. Women (*p*<0.001), rural areas (*p*<0.001), married persons (*p*<0.001), low education level (*p*<0.001), low income (*p*<0.001), and older age (*p*<0.001) were higher in the T3 group. The monthly drinking (*p*<0.001), current smoking (*p*<0.001), and mental stress rates (*p*<0.001) were the highest in the T1 group. The overall average SRH scores and EQ-5D index were 3.08 points and 0.94 points, respectively. ABSI and SRH (*r* = -0.161, *p*<0.001) and ABSI and EQ-5D (*r* = -0.229, *p*<0.001) showed a negative correlation. However, SRH and EQ-5D scores (*r* = 0.433, *p*<0.001) were positively correlated. The overall effect of the independent variable ABSI on the dependent variable EQ-5D was -0.959. SRH partially mediated the effect of ABSI on EQ-5D (indirect effect coefficient = –0.200). These results can be used as basic data to develop strategies and programs to improve health-related quality of life by adjusting ABSI and SRH.

## Introduction

Body shape is a basic component of appearance, while the standard of ideal body shape has changed according to the social situations and cultural environments of that era. Modern society accepts a thin body as an ideal body shape due to the influence of mass media [1]. However, rapid socioeconomic growth in Korea since the 1960s has led to changes in lifestyle and eating habits, and the prevalence of obesity has increased [2]. According to the World Health Organization (WHO), overweight and obesity are ranked fifth as worldwide causes of death [3]. Body mass index (BMI) is known to correlate well with body fat [4, 5] and is commonly used to measure obesity, but it does not indicate body shape [6]. Waist circumference (WC) is widely used as a measure of central adiposity. Several studies have found that WC predicts mortality risk better than BMI [7, 8]. However, WC is highly correlated with BMI, limiting its utility beyond BMI [9]. Therefore, there is a need for a new indicator that can predict body fat and mortality risk to overcome the limitations of existing anthropometric measures. The A Body Shape Index (ABSI) is based on the normalization of WC to BMI and height [10]. A high ABSI indicates that the WC is greater than expected for a given height and weight, corresponding to a more central concentration of body mass. ABSI also predicts mortality risk independent of BMI [11].

Life expectancy in Koreans increased from 62.3 years in 1970 to 83.3 years in 2019 [12]. This quantitative lifespan extension has induced an increase in the prevalence of chronic diseases, and an evaluation index for health status has become necessary. Among several health-related quality of life (HRQL) tools, EuroQol (EQ)-5D is one of the most used tools because it expresses mortality and perfect health as a single numerical value (0 and 1, respectively) [13]. HRQL decreases with increasing levels of obesity, and such decreased scores were noted even in individuals without chronic diseases known to be associated with obesity [14].

Although self-rated health (SRH) is simply evaluated as a single question, it has been widely used as a major index of an individual’s health status. It predicts the mortality rate of various diseases, even after adjusting for various variables [15]. ABSI and SRH have a negative relationship [16], whereas SRH has a positive effect on EQ-5D [17]. Therefore, this study aimed to examine the relationship between ABSI and HRQL; determine the mediating effect of SRH in Korean adults using the seventh (2016-2018) Korea National Health and Nutrition Examination Survey (KNHANES); and provide basic data for developing strategies and programs to improve HRQL.

## Materials and methods

### Participants

This study used data from the 7th (2016-2018) KNHANES conducted by the Korea Disease Control Prevention Agency of the Ministry of Health and Welfare. The participants were selected using a stratified multi-stage clustered complex sampling method based on the area of residence, sex, and age, and the same survey district and household were surveyed every year for three years. Among the 18,670 participants aged ≥ 20 years, pregnant or breast-feeding women (n = 161), those with implausible dietary intake (<500 kcal or >5,000 kcal/day; n = 2,552) [18], those with missing demographic data (n = 1,840), and those without physical characteristics (n = 736) were excluded. Finally, 13,381 participants were included in the analysis. The Institutional Review Board of the Korea Disease Control and Prevention Agency approved the KNHANES (IRB no. 2018-01-03-P-A). Written informed consent was obtained from all participants. The study follows the ethical standards of the Helsinki Declaration 1975.

### Independent variable

ABSI is a metric for assessing the health implications of a given body height, weight, and WC. ABSI was calculated using the formula from a previous study [10]. In this study, using the rank variable generation function of the SPSS program, ABSI was classified into tertile groups (T1 = 0 to <33.3 percentile; T2 = 33.3 to <66.6 percentile; T3 = 66.6-100.0 percentile) based on previous studies [16]. And The higher the tertile group, the greater the risk.

$$\begin{array}{c} A\;\text{Body}\;\text{Shape}\;\text{Index}\;(\text{ABSI}\;)=\text{Waist}\;\text{circumference}\;/(BMI\times 2/3)\times (height\times 1/2) \end{array}$$ (1)

### Dependent variable

The EQ-5D index is a conversion of the five dimensions of the EQ-5D into a single index [19]. The index score of 1 is typically interpreted as the best possible health index. Third, SRH was measured on a 5-point scale with a single question (1 = very bad, 2 = bad, 3 = fair, 4 = good, and 5 = very good).

### Mediating variable

Self-rated health was measured on a 5-point scale (1 = very bad, 2 = bad, 3 = fair, 4 = good, and 5 = very good).

### Control variables

Demographic variables included sex, residential area, marital status, education level, household income, and age. Residential areas were divided into urban and rural areas, marital status was classified as married or unmarried, and education level was classified as elementary school graduate or less, middle school graduate, high school graduate, and college graduate or higher. Household income was also divided into four groups: lowest, lower-middle, upper-middle, and highest quartiles of monthly household income. Health-related factors included monthly drinking rate, current smoking rate, mental stress rate, waking (days/week), strength exercise (days/week), number of chronic diseases, and energy intake. The number of chronic diseases was based on the current prevalence of hypertension, dyslipidemia, diabetes, stroke, cardiovascular disease, chronic kidney disease, and bone disease. Energy intake was analyzed using the 24-h recall method.

### Statistical analysis

All data were analyzed using SPSS (version 23.0; IBM Inc., Chicago, IL, USA) and SPSS Process Macro program (version 4.0; Andrew F. Hayes). Differences in the variables according to ABSI were evaluated using one-way ANOVA for continuous variables (confirmation of normality of dependent variable with Kolmogorov-Smirnov test) and the *x*^2^ test for categorical variables. Pearson’s correlation coefficient analysis was conducted to determine the relationships among the variables. Regression analysis and bootstrapping methods were used to investigate the mediating effect of subjective health status on the relationship between ABSI and EQ-5D. The control variables were adjusted for in the analyses. Statistical significance was set at *P*<0.05.

## Results

### Demographic characteristics

Table 1 shows the demographic characteristics of participants according to the ABSI. The proportion of women and the proportion of rural residences increased in the T2 and T3 groups compared with the T1 group. The proportion of participants who stayed married was higher in the T3 group (93.6%) than in the T1 group (73.5%), whereas at the educational level, the proportion of participants with less than elementary school education was higher in the T3 group, whereas the proportion of those who graduated from college or higher was higher in the T1 group. Furthermore, the T3 group had the lowest household income. The age of participants increased from the T1 group (43.09 years) to the T3 group (62.05 years). Moreover, ABSI was significantly associated with all demographic characteristics (*p*<0.001).

**Table 1:** Comparison of demographic characteristics of participants among the ABSI tertile.

Variables	Total	ABSI^1)^			*P*-value^2)^
	(N = 13,381)	T1 (N = 4,460)	T2 (N = 4,460)	T3 (N = 4,461)	
Sex					
Male	5,612 (41.9)	2,320 (52.0)	1,823 (40.9)	1,469 (32.9)	<0.001
Female	7,769 (58.1)	2,140 (48.0)	2,637 (59.1)	2,992 (67.1)	
Age (years)	52.52±16.60	43.09±13.76*a*^3)^	52.43±15.07^*b*^	62.05±15.18^*c*^	<0.001
20-29	1,388 (10.4)	859 (19.3)	364 (8.2)	165 (3.7)	
30-49	4,463 (33.4)	2,187 (49.0)	1,522 (34.1)	754 (16.9)	<0.001
50-64	3,815 (28.5)	1,088 (24.4)	1,479 (33.2)	1,248 (28.0)	
65	3,715 (27.8)	326 (7.3)	1,095 (24.6)	2,294 (51.4)	
Residence					
Urban	10,873 (81.3)	3,754 (84.2)	3,711 (83.2)	3,408 (76.4)	<0.001
Rural	2,508 (18.7)	706 (15.8)	749 (16.8)	1,053 (23.6)	
Marital status					
Married	11,378 (85.0)	3,277 (73.5)	3,924 (88.0)	4,177 (93.6)	<0.001
Unmarried	2,003 (15.0)	1,183 (26.5)	536 (12.0)	284 (6.4)	
Education level					
≥Primary school	3,412 (25.5)	552 (12.4)	1,087 (24.4)	1,773 (39.7)	
Middle school	1,280 (9.6)	363 (8.1)	420 (9.4)	497 (11.1)	<0.001
High school	4,003 (29.9)	1,605 (36.0)	1,344 (30.1)	1,054 (23.6)	
≥College	4,686 (35.0)	1,940 (43.5)	1,609 (36.1)	1,137 (25.5)	
Household income					
Low	2,699 (20.2)	461 (10.3)	726 (16.3)	1,512 (33.9)	<0.001
Middle low	3,285 (24.5)	1,048 (23.5)	1,076 (24.1)	1,161 (26.0)	
Middle high	3,560 (26.6)	1,395 (31.3)	1,260 (28.3)	905 (20.3)	
High	3,837 (28.7)	1,556 (34.9)	1,398 (31.3)	883 (19.8)	

Values are presented as N (%) or mean±standard deviation (SD).

^1)^ ABSI, A Body Shape Index, divided into tertile groups.

^2)^ P-values were calculated using the *x*^2^ test or one-way ANOVA.

^3)^ Superscript(a, b and c) letters mean that each group in different from the other in Duncan’s test (*P*<0.005).

### Health-related factors

The monthly drinking, current smoking, and mental stress rates were 52.5%, 16.3%, and 26.2%, respectively (Table 2). According to the ABSI group, the proportion of drinking (*p*<0.001), smoking (*p*<0.001), and stress (*p*<0.001) was relatively higher in the T1 group than in the T2 and T3 groups. The number of chronic diseases was also the lowest in the T1 group and increased in the T2 and T3 groups (*p*<0.001).

**Table 2:** Comparison of health-related factors among the ABSI tertile groups.

Variables	Total	ABSI^1)^			*P*-value^2)^
	(N = 13,381)	T1 (N = 4,460)	T2 (N = 4,460)	T3 (N = 4,461)	
Monthly drinking rate	7,009 (52.5)	2,756 (61.9)	2,418 (54.4)	1,835 (41.3)	<0.001
Current smoking rate	2,169 (16.3)	923 (20.7)	657 (14.8)	589 (13.3))	<0.001
Mental stress rate	3,492 (26.2)	1,329 (29.8)	1,114 (25.1)	1,049 (23.7)	<0.001
Chronic disease^3)^					
0	8,256 (61.7)	3,434 (77.0)	2,779 (62.3)	1,994 (44.7)	<0.001
1	2,315 (17.3)	549 (12.3)	789 (17.7))	1,004 (22.5)	
2	1,606 (12.0)	312 (7.0)	526 (11.8)	776 (17.4)	
3	1,204 (9.0)	165 (3.7)	366 (8.2)	687 (15.4)	
Exercise					
Waking day/week	3.50±2.02	3.70±1.98*a*^4)^	3.55±2.03^*b*^	3.26±2.02^*c*^	<0.001
Strength exercise day/week	1.75±1.54	1.90±1.63^*a*^	1.79±1.56^*b*^	1.56±1.40^*c*^	<0.001
Energy intake (kcal)^5)^	1,902.3±785.0	2,056.6±840.3^*a*^	1,922.1±777.7^*b*^	1,728.3±695.4^*c*^	<0.001

Values are presented as N (%) or mean±standard deviation (SD).

^1)^ ABSI, A Body Shape Index, divided into tertile groups.

^2)^*P*-values were calculated using the *x*^2^ test or one-way ANOVA.

^3)^ The number of chronic diseases was based on the current prevalence of hypertension, dyslipidemia, diabetes, stroke, cardiovascular disease, chronic kidney disease, and bone disease.

^4)^ Superscript(a, b and c) letters mean that each group in different from the other in Duncan’s test (*P*<0.005).

^5)^ Energy intake was analyzed using the 24-h recall method.

### Characteristics and correlation of ABSI, SRH, and EQ-5D index

**Fig 1:**
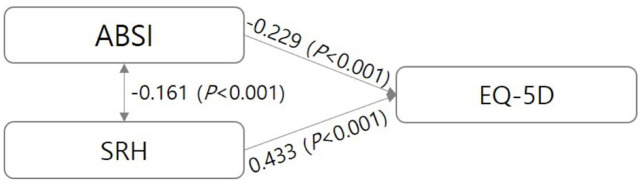
Correlation coefficient of major variables.

Table 3 shows the differences in ABSI, SRH, and EQ-5D scores according to the ABSI. The ABSI scores of the T1, T2, and T3 groups were 0.059, 0.064, and 0.070, respectively. In terms of SRH, the T1 group had the highest score (3.22/5), whereas the T3 group had the lowest score (2.90/5, *p*<0.001). Moreover, the EQ-5D score of the T1 group was 0.97/1, which was lower than 0.95/1 of the T2 group and 0.91 of the T3 group (*p*<0.001). The absolute value of skewness ranged from 0.018 to 1.648, which was less than 3.0. The absolute value range of the kurtosis was -0.027 to 2.987, which was confirmed to be less than 10, and normality was satisfied [20]. Fig 1 shows the correlations between ABSI, SRH, and EQ-5D scores. ABSI and SRH (*r* = -0.161, *p*<0.001) and ABSI and EQ-5D (*r* = -0.229, *p*<0.001) showed a negative correlation, whereas SRH and EQ-5D (r = 0.433, *p*<0.001) showed a positive correlation.

**Table 3:** Comparison of health-related factors among the ABSI tertile groups.

Variables	Total	ABSI^1)^			*P*-value^2)^	Skewness	Kutrosis
	(N = 13,381)	T1 (N = 4,460)	T2 (N = 4,460)	T3 (N = 4,461)			
ABSI	0.064±0.005	0.059±0.002*a*^3)^	0.064±0.001^*b*^	0.070±0.003^*c*^	<0.001	1.648	2.987
SRH	3.08±0.86	3.22±0.81^*a*^	3.11±0.84^*b*^	2.90±0.90^*c*^	<0.001	0.018	-0.027
EQ-5D	0.94±0.11	0.97±0.08^*a*^	0.95±0.10^*b*^	0.91±0.14c^c^	<0.001	0.982	-1.011

Values are presented as N (%) or mean±standard deviation (SD).

^1)^ ABSI, A Body Shape Index, divided into tertile groups.

^2)^*P*-values were calculated using the one-way ANOVA.

^3)^ Superscript(a, b and c) letters mean that each group in different from the other in Duncan’s test (*P*<0.005).

### Verification of the mediating effect

In step 1, the verification results for the mediation model from ABSI to EQ-5D through SRH are shown in Table 4. The overall effect of ABSI on SRH was -5.008, indicating that the higher the ABSI, the lower the SRH, and the influence was 19.0% (F = 187.975, *p*<0.001). In step 2, the overall effect of the independent variable ABSI on the dependent variable EQ-5D was -1.190, indicating that the higher the ABSI, the lower the EQ-5D, and the effect was 23.6% (F = 248.498, p<0.001). In step 3, the overall effect of the independent variable ABSI on the dependent variable EQ-5D was -0.959, which was lower than -1.190 when no parameters were considered. This shows that SRH, as a parameter, partially mediates the effect of the dependent variable EQ-5D. Moreover, the coefficient of the indirect effect of ABSI on EQ-5D was -0.200 (= -5.008 × 0.040).

**Table 4:** Verification results for the mediation model of the ABSI, EQ-5D, and SRH scores.

Stage	Dependent variable	Independent variable	B	SE^1)^	*β*	t	*P*-value^2)^
Stage 1	Self-rated health	(constant)	3.209	0.130		24.715	<0.001
		ABSI^3)^	-5.008	1.885	-0.034	-3.021	<0.01
			R^2^ = 0.190, F = 187.975 (*P* <0.001)				
Stage 2	EQ-5D	(constant)	1.002	0.017		58.041	<0.001
		ABSI	-1.190	0.250	-0.052	-4.751	<0.001
			R^2^ = 0.236, F = 248.498 (*P* <0.001)				
Stage 3	EQ-5D	(constant)	0.871	0.017		51.2867	<0.001
		Self-rated health	0.040	0.001	0.296	30.052	<0.001
		ABSI	-0.959	0.239	-0.042	-4.020	<0.001
			R^2^ = 0.307, F = 326.314 (*P*<0.001)				

Control variables: sex, residence, marital status, education level, income, age, drinking, smoking, mental stress, chronic disease, exercise, and energy intake in all participants.

^1)^ SE, standard error.

^2)^*P*-value was calculated using the t-test or F-test.

^3)^ ABSI, A Body Shape Index.

**Fig 2:**
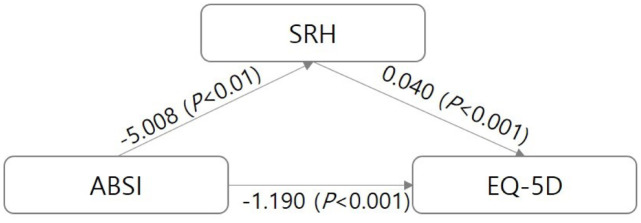
Statistical model for research results.

In the process of evaluating ABSI’s effect on EQ-5D, bootstrapping confirmed whether -0.200, which is the coefficient of the mediating effect of subjective health status, was significant (Table 5). The number of samples resampled for bootstrapping was 5,000, and the upper and lower limits of the mediating effect coefficient obtained at the 95% confidence interval were -0.3865 and -0.0759, respectively, which was also significant. Therefore, the higher the ABSI, the lower the SRH, and the mediating pathway that leads to EQ-5D is significant. The direct effect of ABSI on EQ-5D was also significant. Therefore, the partial mediation model is supported. The results of the above analysis are presented as a model in Fig 2.

**Table 5:** Bootstrapping results for the mediating effects of self-rated health.

Variable	Effect	S.E.^1)^	95% CI^2)^	
			LLCI^3)^	ULCI^4)^
Self-rated health	-0.200	0.0800	-0.3865	-0.0759

^1)^ SE, standard error.

^2)^ 95% confidence interval.

^3)^ Lower limit of confidence interval.

^4)^ Upper limit of confidence interval.

## Discussion

As obesity has emerged as a major cause of premature death worldwide [3], the ABSI, developed as an index that can predict mortality while complementing the shortcomings of the existing obesity index, is independent of BMI [10]. A study reported that a standard deviation increase in ABSI was associated with an increase in the odds of hypertension, type 2 diabetes, cardiovascular disease, and all-cause mortality risk. The ABSI also outperformed BMI and WC in predicting all-cause mortality [21]. Therefore, this study aimed to investigate the relationship between the ABSI, a newly proposed body type index for Korean adults, and the EQ-5D index using data from the KNHANES and to confirm the mediating effect of SRH. Regarding the ABSI, the standard for predicting diseases has not yet been established, but the overall average ABSI in this study was 0.064, which was lower than that in the Korean adult study (ABSI = 0.075) [22]. Furthermore, Shin stated that the reference value of ABSI for predicting the risk of metabolic syndrome in Korean adults was 0.076, whereas the average ABSI of our T3 group was 0.070, which is lower than the reference value.

Among health-related factors, drinking, smoking, and mental stress rates are obesity-related factors [23, 24]. In our study, the monthly drinking rate, current smoking rate, and mental stress rate were the highest in the T1 group among the tertile groups. According to the 2018 KNHANES [25], the monthly drinking, current smoking, and mental stress rates decreased as the number of people in their 40s, 50s, and 60s decreased. This may be related to the fact that the average age was 43.09, 52.43, and 62.05 years in the T1, T2, and T3 groups, respectively. Although drinking is a factor that positively affects obesity [23], obesity-related drinking patterns include frequency of drinking, amount of drinking, and frequency of binge drinking. It is well known that smokers have a lower obesity rate than non-smokers [24]; therefore, it can be explained that the ratio of the monthly drinking and current smoking rates was the highest in our T1 group.

The mental stress rate in this study conflicts the findings of a previous report [24] stating that both obesity and abdominal obesity rates were higher in the stress-cognitive group and that appearance stress has a positive correlation with obesity stress [26]. This seems to be the effect of age, because the T1 group is in their 40s with active social activities, the T2 group is in their 50s preparing for retirement, and the T3 group is in their retirement period. An increase in the ABSI increases the risk of chronic diseases [21]. In our study, the prevalence of chronic diseases was higher in the T3 group than in the T1 group. Exercise is also a factor that positively affects body shape maintenance and reduction by increasing energy consumption, and the prevalence of walking and strength exercises was the highest in our T1 group. High energy intake is an important factor related to obesity, and the highest energy intake was observed in our T1 group. This result seems to be related to the overreporting of energy intake by underweight people and the underreporting of energy intake by obese people [27], as seen in previous reports.

Awareness of SRH is a representative indicator of an individual’s overall health status. SRH is known to be affected by socioeconomic indicators such as age, education level, employment status, and health-related lifestyle habits (such as smoking, drinking, exercise, stress, and obesity) [16, 28]. The average SRH score in our study (3.08) was lower than that 3.41 reported in a previous study using data from South Korea, China, Japan, and Taiwan [28]. According to the ABSI tertile, the SRH score in the T2 and T3 groups decreased significantly compared with that in the T1. Especially, the ABSI T3 group showed elderly age, low education level, low household income, high prevalence of chronic diseases, and low walking practice rate, which are known to be factors that lower SRH. In the correlation analysis, the ABSI and SRH were negatively correlated. This seems to be related to the fact that the higher the ABSI, the less exercise and the higher the prevalence of chronic diseases.

HRQL refers to a state of health that each patient feels subjectively, including physical, mental, and social functions, and obesity has a negative effect on the quality of life [29–31]. In our study, the overall mean EQ-5D score, was 0.94/1, and according to the ABSI tertile, there was a significant decrease in the EQ-5D score among the T1, T2, and T3 groups, suggesting a negative correlation between ABSI and EQ-5D. This may be related to the increase in the prevalence of chronic diseases as the ABSI value increases, similar to that noted for SRH. It has been reported that the perception of subjective health status has a positive relationship with quality of life [17]. In our study, SRH and EQ-5D showed a positive correlation.

The mediating effect test explains how and why the parameters are related when the independent variable affects the dependent variable [32]. In our study, SRH partially mediated the effect of the ABSI on EQ-5D scores. In other words, compared with the direct effect of the ABSI on EQ-5D score, which is an HRQL parameter, the effect of the ABSI was reduced when SRH was considered. This supports the results of previous studies [16, 17, 28].

This study had several limitations. First, since cross-sectional data were used, the causal sequence between the independent and dependent variables could not be determined. Second, domestic studies on the ABSI are insufficient, and in particular, previous studies on the difference between SRH and EQ-5D scores according to tertiles are limited. Thus, the interpretation of the study results is limited, and various follow-up studies including factors such as SRH, EQ-5D, chronic disease, and diet using the ABSI as an independent variable for Koreans are needed. Nevertheless, this study has the advantage of verifying the mediating effect of SRH in the relationship between the ABSI, a new body shape index, and EQ-5D, and generalizing the results by targeting 13,381 people extracted through stochastic sampling.

## Conclusions

In order for the newly proposed ABSI to become a valid index, studies evaluating various aspects are needed. As a result of the first question in this study, we demonstrated that ABSI is negatively related to EQ-5D. Therefore, in order to improve the quality of life, it is necessary to have an appropriate weight and waist circumference that affect the ABSI value. And we found that SRH plays a mediating role in the relationship between ABSI and EQ-5D. These results suggest that a strategy to increase SRH by lowering ABSI can improve quality of life. In addition, further studies on ABSI diagnostic criteria for Koreans are needed. Results of this study will be used as data for developing strategies and programs to improve HRQL.

## References

[1] Son KJ, Lee MS. The effects of sociocultural attitude toward appearance on perceptual, attitudinal body images and clothing behaviors. J Korean Home Economics Assoc. 2009 Feb;47(2):97–110. uci: G704-000012.2009.47.2.002

[2] Kang NE, Kim SJ, Oh YS, Jang S. The effects of body mass index and body shape perceptions of South Korean adults on weight control behaviors. Nutr Res Pract. 2020 Apr;14(2):160–166. doi: 10.4162/nrp.2020.14.2.160 32256991 PMC7075741

[3] World Health Organization. Available online: https://apps.who.int/iris/handle/10665/44203 (Dec.16.2022).

[4] Janjic J, Baltic ZM, Glisic M, Ivanovic J, Boskovic M, Popovic M, et al. Relationship between body mass index and body fat percentage among adoles¬cents from Serbian Republic. Child Obes. 2016 May;1(2):1–5. doi: 10.21767/2572-5394.100009

[5] Ramel A, Halldorsson TI, Tryggvadottir EA, Martinez JA, Kiely M, Bandarra NM, et al. Relationship between BMI and body fatness in three European countries. Eur J Clin Nutr. 2013 Jan;67(3):254–258. doi: 10.1038/ejcn.2013.6 23361157

[6] González AS, Bellido D, Buño MM, Pértega S, Luis DD, Martínez-Olmos M, et al. Predictors of the metabolic syndrome and correlation with computed axial tomography. Nutr. 2007 Jan;23(1):36–45. doi: 10.1016/j.nut.2006.08.019 17189089

[7] Simpson JA, MacInnis RJ, Peeters A, Hopper JL, Giles GG, English DR. A comparison of adiposity measures as predictors of all-cause mortality: the Melbourne Collaborative Cohort Study. Obes. 2007 Apr;15(4):994–1003. doi: 10.1038/oby.2007.622 17426335

[8] Kuk JL, Ardern CI. Influence of age on the association between various measures of obesity and all-cause mortality. J Am Geriatr Soc. 2009 Nov;57(11):2077–84. doi: 10.1111/j.1532-5415.2009.02486.x 19754497

[9] Lee GS, Choi SG, Park SM. Association of waist circumference with muscle and fat mass in adults with a normal body mass index. Nutr Res Pract. 2021 Oct;15(5):604–612. doi: 10.4162/nrp.2021.15.5.604 34603608 PMC8446684

[10] Krakauer NY, Krakauer JC. A new body shape index predicts mortality hazard independently of body mass index. PLOS ONE. 2012 Jul;7(7):e39504. doi: 10.1371/journal.pone.0039504 22815707 PMC3399847

[11] Dhana K, Kavousi M, Ikram MA, Tiemeier HW, Hofman A, Franco OH. Body shape index in comparison with other anthropometric measures in prediction of total and cause-specific mortality. J Epidemiol Community Health. 2016 Jan;70(1):90–6. doi: 10.1136/jech-2014-205257 26160362

[12] Korean Statistical Information Service. Available online: https://kosis.kr (Dec.16.2022).

[13] Balestroni G, Bertolotti G. EuroQol-5D (EQ-5D): an instrument for measuring quality of life. Monaldi Arch Chest Dis. 2012 Sep;78(3):155–9. doi: 10.4081/monaldi.2012.121 23614330

[14] Sach TH, Barton GR, Doherty M, Muir KR, Jenkinson C, Avery AJ. The relationship between body mass index and health-related quality of life: comparing the EQ-5D, EuroQol VAS and SF-6D. Int J Obes. 2007 Jan;31(1):189–96. doi: 10.1038/sj.ijo.0803365 16682976

[15] Idler EL; Benyamini Y. Self-rated health and mortality: a review of twenty-seven community studies. J Health Soc Behav. 1997 Mar;38(1):21–37. doi: 10.2307/2955359 9097506

[16] Jang SK, Kim JH. A study on the relationship between a body shape index and poor self-rated health. Health Society Welfare Review. 2018 Sep;38(3):109–28. doi: 10.15709/hswr.2018.38.3.109

[17] Oh HS. Important significant factors of health-related quality of life (EQ-5D) by age group in Korea based on KNHANES. J Korean Data & Infor Sci Soci. 2017 May;28(3):573–084. doi: 10.7465/jkdi.2017.28.3.573

[18] Willett W. Nutritional epidemiology, 3rd ed, United Kingdom: NY, USA, 2013.

[19] Rabin R, Charro FD EQ-5D: a measure of health status from the EuroQol Group. Ann Med. 2001 Jul; 33(5):337–343. doi: 10.3109/07853890109002087 11491192

[20] Kline PB Principles and practice of structural equation modeling, 3rd ed, GuilfordPress: NY, USA, 2010.

[21] Ji M, Zhang S, An R. Effectiveness of A Body Shape Index (ABSI) in predicting chronic diseases and mortality: a systematic review and meta-analysis. Obes. Rev. 2018 May; 19(5):737–759. doi: 10.1111/obr.12666 29349876

[22] Shin KA. Assessing A Body Shape Index and waist to height ratio as a risk predictor for insulin resistance and metabolic syndrome among Korean adults. Korean J Clin Lab Sci. 2018 Mar;50(1):44–53. doi: 10.15324/kjcls.2018.50.1.44

[23] Ledo YIR, Guerra DA, Patchana Y, Ladisa M, Garcia OV, Miguel HGS, et al. Relationship between alcohol consumption and obesity determinated with different scales. Academic J Health Sci. 2021 Apr;36(2):64–69. doi: 10.3306/AJHS.2021.36.02.64

[24] Kim BY, Lee ES. A new body shape index predicts mortality hazard independently of body mass index. J Korean Public Health Nur. 2017 Dec;31(3):478–91. doi: 10.5932.JKPHN.2017.31.3.478

[25] Ministry of Health and Welfare. Available online: https://www.mohw.go.kr (Dec.16.2022).

[26] Kim YS, Lee HJ. A Study on self-esteem, stress and depression of abdominal obesity control subjects. Asia-pacific J Multimedia Services Conv Art Humanities Socio. 2019 Apr;9(4):437–46. doi: 10.35873/ajmahs.2019.9.4.042

[27] Mertz W, Tsui JC, Judd JT, Reiser S, Hallfrisch J, Morris ER, et al. What are people really eating? The relation between energy intake derived from estimated diet records and intake determined to maintain body weight. Am J Clin Nutr. 1991 Aug;54(2):291–5. doi: 10.1093/ajcn/54.2.291 1858692

[28] Noh J, Kim J, Yang Y, Park J, Cheon J, Kwon YD. Body mass index and self-rated health in East Asian countries: comparison among South Korea, China, Japan, and Taiwan. PLOS ONE. 2017 Aug;12(8):e0183881. doi: 10.1371/journal.pone.0183881 28846742 PMC5573277

[29] Park YW, Shin HC, Kim CH. Health-related quality of life in people with overweight and large waist circumference. J Korean Acad Fam Med. 2000 Jun;21(6):753–61.

[30] Kim JH. Association between body shape index, perceived body shape and self-rated health, quality of life in Korean adult population using sixth Korea National Health and Nutrition Examination Survey. Health Social Welfare Review. 2018 Dec;38(4):323–40. doi: 10.15709/hswr.2018.38.4.323

[31] Kim S, Jo M, Ock M, Lee S. Exploratory study of dimensions of health-related quality of life in the general population of South Korea. J. Prev Med Public Health. 2017 Nov;50(6):361–368. doi: 10.3961/jpmph.16.076 29207449 PMC5717327

[32] Jung SH, Seo DG. Assessing mediated moderation and moderated mediation: guidelines and empirical illustration. Korean J Psycology: General. 2016 Mar;35(1):257–82. doi: 10.22257/kjp.2016.03.35.1.257

